# Partial Cranial Cruciate Ligament Tears Treated with Stem Cell and Platelet-Rich Plasma Combination Therapy in 36 Dogs: A Retrospective Study

**DOI:** 10.3389/fvets.2016.00112

**Published:** 2016-12-14

**Authors:** Sherman O. Canapp, Christopher S. Leasure, Catherine Cox, Victor Ibrahim, Brittany J. Carr

**Affiliations:** ^1^Veterinary Orthopedic & Sports Medicine Group, Annapolis Junction, MD, USA; ^2^Regenerative Orthopedic & Sports Medicine, Washington, DC, USA

**Keywords:** bone marrow aspirate concentrate, adipose-derived progenitor cells, platelet-rich plasma, cranial cruciate ligament tear, anterior cruciate ligament tear, mesenchymal stem cells, regenerative therapy

## Abstract

**Objective:**

To evaluate outcomes in 36 dogs with a partial cranial cruciate ligament (CCL) tear treated with autologous bone marrow aspirate concentrate (BMAC) or adipose-derived progenitor cells (ADPC) with platelet-rich plasma (PRP) combination.

**Materials and methods:**

Medical records of client-owned dogs diagnosed with an early partial (≤50%) tear of the craniomedial band of the CCL that was treated with BMAC–PRP or ADPC–PRP were reviewed from 2010 to 2015. Signalment, medical history, physical and orthopedic examination, objective temporospatial gait analyses, radiographs, day 0 and day 90 diagnostic arthroscopy findings, treatment, and outcome were among the data collected. A functional owner questionnaire, including the validated Helsinki chronic pain index (HCPI), was sent to owners whose dog was known to not have had a tibial plateau leveling osteotomy (TPLO). Statistical analysis was performed on data, where significance was established at *p* < 0.05.

**Results:**

Stifle arthroscopy findings at 90 days posttreatment were available on 13 of the 36 dogs. In nine dogs, a fully intact CCL with marked neovascularization and a normal fiber pattern was found with all previous regions of disruption healed. One dog revealed significant improvement and received an additional injection. The remaining three dogs had a >50% CCL tear, and a TPLO was performed. Four additional dogs were known to have had a TPLO performed elsewhere. Baseline and day 90 posttreatment objective gait analyses were available on 11 of the 36 dogs. A significant difference was found between the treated limb total pressure index percent (TPI%) at day 0 and day 90 (*p* = 0.0124), and between the treated limb and contralateral limb TPI% at day 0 (*p* = 0.0003). No significant difference was found between the treated limb and contralateral limb TPI% at day 90 (*p* = 0.7466). Twelve questionnaires were returned, of which eight were performance/sporting dogs. Seven of the eight had returned to sport; the remaining dog had just begun a return to sport conditioning program 6 months posttreatment. All 12 respondents believed that their dog had an excellent or very good quality of life and rated their dog’s procedural outcome as excellent or good.

**Conclusion:**

The use of BMAC–PRP and ADPC–PRP shows promise for the treatment of early partial CCL tears in dogs. Further studies are needed and should be randomized, blinded, and controlled.

## Introduction

The cranial cruciate ligament (CCL) is necessary to stabilize the stifle joint in dogs; however, its rupture is one of the most common causes of hind limb lameness and the most common stifle joint diseases in dogs (1–3). Surgical intervention is recommended to correct the instability, restore the stifle to function, and delay the onset of osteoarthritis. Although no specific surgical procedure has been established as the gold standard in veterinary medicine, intracapsular, extracapsular, and osteotomy techniques are often used (4–9). Short- and long-term complications can occur, including the progression of osteoarthritis (6, 10–19). While these techniques are typically utilized for an unstable stifle, or a complete tear, questions still remain as to how to proceed with an early partial tear where a functional, stable stifle remains. Although an early partial tear is likely to progress to a complete tear if left untreated due to the degenerative cascade of effects that occurs, the benefits of surgery may not outweigh the risks. Therefore, an alternative treatment option could be considered.

Mesenchymal stem cell (MSC) therapy is a more recent cell-based technique that is being utilized in numerous orthopedic conditions. Multipotent progenitor cells and MSCs are two types of adult stem cells used for tissue regeneration and their trophic effects (20). Found from various sources, including bone marrow and adipose tissue, MSCs and progenitor cells have the potential to differentiate into multiple cell lineages including bone, cartilage, and connective tissue such as ligaments (21, 22). Several studies report that MSCs are an optimal source for ligament regeneration due to their high proliferation and collagen production potential and ability to quickly differentiate into ligament fibroblasts (23, 24).

Numerous animal studies have been conducted to evaluate the regeneration potential of MSCs, which have revealed that MSCs result in cell engraftment in the CCL, meniscus, and cartilage when injected intra-articularly (25, 26). Another study found that intra-articularly injected MSCs can accelerate the healing of partial anterior cruciate ligament (ACL) tears in rats when evaluated biomechanically and histologically (27). In this study, in treatment group at 2 and 4 weeks after partial transection of the ACL, the transected area was covered with healing tissues in which GFP-positive cells were detected (27). The treatment group also had better histologic scores at 2 and 4 weeks and significantly higher ultimate failure load than the control group 4 weeks following transection (27). Similar results were found in a recent study on rats where 1 week following partial transection of the ACL, they received an intra-articular injection of either fresh bone marrow cells (BMC) or cultured MSCs that were labeled (28). At 4 weeks following injection, the cells were not only located within the ACL but the ACL also appeared both histologically normal and with more mature spindle cells (28). This study also showed that the tensile strength in the BMC group reached near normal levels at 4 weeks after injection and significantly higher levels of transforming growth factor-β1 in the ACL and knee joint fluid in the BMC group when compared to the control group at 4 weeks after injection (28). A recent study was performed on dogs diagnosed with a unilateral unstable CCL rupture that was surgically stabilized, which had a contralateral, stable partial CCL (29). At the time of surgical stabilization of the unstable stifle, bone marrow-derived mesenchymal stem cells (BM-MSCs) were collected, culture expanded, and subsequently injected intravenously and intra-articularly into the contralateral, stable stifle with a partial CCL. Inflammatory markers within synovium and serum were monitored for 8 weeks and showed that both systemic and synovial markers had decreased at 8 weeks postinjection. Thus, this study concluded that intra-articular injection of autologous BM-MSCs had profound effects on the correlation and conditional dependencies of inflammatory markers, which could ameliorate the risk of a second CCL rupture (29). Human clinical studies show similar potential, where patients have shown improvement in objective measures of ACL integrity and subjective outcomes after bone marrow MSC injection of grade 1 through three ACL tears (30). Safety for clinical use has been verified with no neoplastic complications (31).

Platelet-rich plasma (PRP) provides growth factors to enhance and promote MSC engraftment, providing a synergistic effect. Growth factors improve cell proliferation, promote an anabolic phase of cells, and stimulate MSC differentiation into fibroblasts (32–35). Additionally, PRP provides a three-dimensional scaffold for MSCs to facilitate differentiation and cell survival during delivery (36). A recent study showed that several intra-articular injections of PRP alone indicated evidence of CCL repair and remodeling in dogs (37). These findings promote the use of PRP in combination with MSCs to aid in ligament healing.

The purpose of this study was to evaluate the use of autologous bone marrow aspirate concentrate (BMAC) or adipose-derived progenitor cells (ADPC) with PRP combination and a recommended rehabilitation therapy program for the treatment of early partial CCL tears in dogs. Assessing outcomes in dogs has not been previously reported.

## Materials and Methods

### Case Selection

Medical records of client-owned dogs were reviewed for cases diagnosed arthroscopically with an early partial (≤50%) tear of the craniomedial band of the CCL that was treated with BMSC–PRP or ADPC–PRP between January 2010 and December 2015 at Veterinary Orthopedic & Sports Medicine Group (VOSM). Data collected included signalment, medical history, physical and orthopedic examination findings, objective temporospatial gait analysis findings, diagnostic radiography results, arthroscopy findings, treatments, and outcome. Patients with concomitant stifle joint problems, or orthopedic or neurologic conditions affecting the hind limbs, were excluded from analysis.

### Orthopedic Examination

Dogs were assessed at baseline and 30, 60, and 90 days posttreatment with an orthopedic and neurologic examination. Degree of lameness, presence of stifle effusion, presence of cranial drawer and cranial tibial thrust in flexion and extension, and McMurray test result were among the findings recorded (38). Additional abnormal orthopedic or neurologic findings were also recorded.

### Objective Gait Analysis

Objective gait analysis was performed at baseline and 90 days posttreatment in a quiet room using a temporospatial pressure sensing walkway. The walkway system was equipped with an 8.23 m × 0.85 m portable mat with 29,952 encapsulated sensors (GaitFour Pressure Sensing Walkway, platinum version, CIR Systems Inc., Havertown, PA, USA). The active dimensions of the mat were 8.04 m × 0.61 m. A 1.25 m × 0.85 m section of inactive mat was placed at each end of the walkway system to provide transition surfaces when entering and exiting the system. The mat was calibrated by the manufacturer. The walkway system interfaced with a computer and software program for processing and storing raw data recorded from quadruped gait analysis (GaitFour, platinum version, CIR Systems Inc., Havertown, PA, USA). One camera was positioned at a height of 50 cm at the end of the walkway system to record movement (Logitech mega pixel web camera, Logitech, Freemont, CA, USA). Digital video files of each pass across the walkway system were automatically linked to the data files for footfall verification.

Each dog was familiarized with the room prior to walking on the mat and was handled by the same examiner. Dogs were made to walk on the mat until they appeared relaxed, approximately four to six passes per dog. Three passes were recorded at both a walk and a trot, where a pass was defined as a dog moving along the length of the walkway in one direction for four to six gait cycles. A satisfactory pass consisted of minimal head turning, a walk velocity of 0.9–1.2 m/s or trot velocity of 1.8–2.6 m/s, and less than two standard deviations in gait cycle velocity differences. The software program identified the paw print of each footfall and analyzed multiple variables for each pass. The data calculated for each pass provided the total pressure index percent (TPI%) (sum of peak pressure values recorded from each activated sensor by a paw during mat contact/total sum of peak pressure values for all feet × 100). The TPI% of the treated limb was compared to the TPI% of the contralateral limb at baseline and at 90 days posttreatment. The TPI% of the treated limb at day 0 was also compared to the treated limb TPI% at 90 days posttreatment. Dogs diagnosed with bilateral partial CCL tears were excluded from this analysis.

### Radiographs

Routine lateral and craniocaudal radiographs were performed of both stifles in all patients at baseline and evaluated for evidence of stifle osteoarthritis by a board-certified veterinary surgeon (39).

### Diagnostic Stifle Arthroscopy

A standard parapatellar portal was utilized for arthroscopic evaluation of the stifle. The stifle was clipped and aseptically prepared and a 22-gauge needle was placed into the lateral compartment. Synovial fluid was aspirated using a 5 ml syringe to ensure that the needle was in the joint. Approximately 10 ml of sterile saline solution was injected into the stifle to distend the joint. A 15 blade was used to create medial and lateral parapatellar stab incisions to allow insertion of the cannula and trochar into the stifle. A 1.9 or 2.7 mm arthroscope was inserted into the cannula, depending on the size of the dog. The stifle joint was explored arthroscopically and assessed all structures, including the craniomedial and caudolateral band of the CCL, the caudal cruciate ligament, and medial and lateral menisci. Cartilage breakdown was scored using the Outerbridge classification (40). The fat pad was not removed with a shaver, but rather an L-shaped probe (Graduated Black Probe 2.5 mm tip length, Arthrex Vet Systems, Naples, FL, USA) was inserted and used to move the fat pad for direct observation of the CCL. The torn portion (percent) of the CCL was measured using the standardized L probe. If ≤50% of the craniomedial band of the CCL was determined to be damaged, a BMAC–PRP or ADPC–PRP injection was to be performed. If a >50% tear was observed, a tibial plateau leveling osteotomy (TPLO) was performed. The arthroscopic incisions were closed routinely.

### Bone Marrow Collection for BMAC

The dog was placed in lateral recumbency, and the hip was clipped and aseptically prepared. The vaclock syringe and bone marrow aspiration needle were primed with 20 ml of heparin 1,000 U/ml, leaving 5 ml in the vaclock syringe. A 15 blade was used to create a 0.5 cm stab incision at the point of the proximal femur and an 11 gauge × 110 mm bone marrow aspiration needle was inserted into the greater trochanter. Approximately 30 ml of bone marrow was obtained. The incision was closed routinely.

### Blood Collection for PRP with BMAC

The jugular vein was clipped and aseptically prepared. An 18-gauge butterfly needle was used to collect 50 ml of blood into a 60 ml syringe that contained 10 ml of anticoagulant citrate dextrose solution A (ACD-A).

### BMAC and PRP Processing

Bone marrow was processed using a validated system according to manufacturer’s protocols (BMC Stem Cell Kit, Companion Regenerative Therapies, Newark, DE, USA). Briefly, the 150–170-µm filter, 60-ml syringe, and concentrating device were primed with heparin 1,000 U/ml. The vaclock syringe containing the bone marrow was attached to the filter and injected through the filter to the empty 60 ml syringe. The bone marrow was then injected into the concentrating device, placed into the centrifuge (Executive Series Centrifuge II, Companion Regenerative Therapies, Newark, DE, USA), and spun for 10 min at 3,600 RPM. After centrifugation, a 60-ml syringe was used to aspirate the plasma layer. A 12-ml syringe was then used to aspirate 3 ml of BMAC. Approximately 30,000 MSCs were present in the initial 30-ml bone marrow sample (41).

Whole blood was processed using a validated system according to manufacturer’s protocols (PurePRP^®^ Kit, Companion Regenerative Therapies, Newark, DE, USA). Briefly, the syringe containing the blood and ACD-A was injected into the first concentrating device, placed into the centrifuge (Executive Series Centrifuge II, Companion Regenerative Therapies, Newark, DE, USA), and spun for 1 min at 3,600 RPM. After centrifugation, a 60-ml syringe was used to aspirate the platelet plasma suspension layer and was then injected into the second concentrating device. The concentrating device was placed into the centrifuge and spun for 5 min at 3,800 RPM. After centrifugation, a 60-ml syringe was used to aspirate the plasma until the aspirating disc reached the bottom of the tube. A 12-ml syringe was used to inject 10 ml of air into the device to allow for re-suspension of the platelets at the base of the device. The device was swirled to re-suspend the platelets into the remaining plasma. The 12-ml syringe was then used to aspirate 4 ml of PRP.

To confirm the PRP product composition, a baseline blood and PRP product WBC differential, RBC concentration, and platelet concentration were obtained on all dogs using an in-house hematology analyzer that had been calibrated according to manufacturer standards (Idexx LaserCyte Hematology Analyzer, Idexx Laboratories, Westbrook, ME, USA). The PRP obtained a sixfold to sevenfold increase in platelets, 85% reduction in neutrophils, and 95% reduction in red blood cells from whole blood. Platelet, neutrophil, and red blood cell counts were verified prior to use.

### Adipose Collection for ADPC

The abdomen was clipped and aseptically prepared. A 4-cm incision was made in midline in the cranial abdomen, and a 2-cm incision was made through the linea. The falciform ligament was identified. Approximately 20 g of falciform adipose tissue was placed into a sterile container with Dulbecco’s modified Eagle’s medium (DMEM) supplemented with fetal bovine serum, penicillin, and streptomycin. The incision was closed routinely.

### Blood Collection for PRP with ADPC

The jugular vein was clipped and aseptically prepared. An 18-gauge butterfly needle was used to collect 25 ml of blood into a 30 ml syringe that contained 5 ml of citrate phosphate dextrose adenine anticoagulant.

### ADPC and PRP Processing

Adipose and blood samples were shipped in validated shipping boxes at 4°C to the Virginia Tech Marion DuPont Scott Equine Medical Center Regenerative Medicine Service for processing. ADPC and ACS were processed using their proprietary protocols. Briefly, adipose was mechanically and enzymatically separated to release ADPCs. The cells were then washed in phosphate-buffered saline (PBS), and nucleated cells were counted and cultured in stem cell media at 37°C in a 5% CO_2_ incubator with 95% humidity. Cells were washed in PBS once at 70% confluency, trypsinized, and suspended in PRP. Approximately 5 million ADPCs were present in the sample, which were verified prior to suspension. PRP was prepared *via* centrifugation to obtain a fourfold increase in platelets and 80% reduction in white blood cells from whole blood. Platelet and white blood cells counts were verified prior to use. The ADPC–PRP was shipped to VOSM in validated shipping boxes at 4°C for subsequent injection.

### Intra-Articular Injection of BMAC–PRP or ADPC–PRP

Bone marrow aspirate concentrate and PRP were drawn into an empty syringe in a 1:1 ratio *via* a three-way stopcock. The stifle was clipped (if ADPC–PRP) and aseptically prepared. A 22-gauge needle was placed into the lateral compartment of the stifle and synovial fluid was aspirated using a 5-ml syringe to ensure the needle was in the joint. Approximately 2–4 ml of BMAC–PRP or 1–2 ml of ADPC–PRP was injected intra-articularly into the stifle; volume depended on the size of the dog. If the dog was below 15 kg in weight, they received 2 ml of BMAC–PRP or 1 ml of ADPC–PRP. If the dog was above 15 kg in weight, they received 4 ml of BMAC–PRP or 2 ml of ADPC–PRP. All dogs were placed into a soft-padded bandage for 12–24 h. Once the soft-padded bandage was removed, dogs were placed into a custom stifle orthotic that had been previously molded and constructed (Animal Orthocare, LLC, Chantilly, VA, USA) if the owner was concerned about their ability to confine and control their dog or if increased laxity had been noted on palpation at the initial examination. All owners were instructed to discontinue NSAIDs, corticosteroids, or anti-inflammatory supplements at least 2 weeks prior to injection. Owners were given the option to choose between BMAC–PRP and ADPC–PRP. The option to bank culture-expanded ADPCs and blood at the Virginia Tech Marion DuPont Scott Equine Medical Center was conveyed to the owner, while BMAC would not typically be banked. BMAC–PRP treatment would be performed the same day as stifle arthroscopy, while ADPC–PRP treatment would occur approximately 2 weeks after being sent for culture, which may play into the decision for owners that traveled a long distance to VOSM. Following the collection procedure and/or injection, patients were placed on tramadol (3–4 mg/kg by mouth every 8 h) or codeine (1–2 mg/kg by mouth every 8 h) for 3 days. The cost of each treatment was equal.

### Posttreatment Rehabilitation Therapy

All patients were instructed to enroll in formal rehabilitation therapy following treatment consisting of once weekly manual/massage therapy, class IIIb low-level laser therapy for the affected stifle (5 J/cm^2^) and a twice daily at home exercise program for the first 8 weeks. NSAIDs, corticosteroids, anti-inflammatory supplements, class IV low level laser therapy, therapeutic ultrasound, hydrotherapy, and TENS/NMES were not permitted for the first 8 weeks posttreatment as their effect on stem cell and PRP therapy is still not fully known.

### Validated Functional Questionnaire

An owner questionnaire, including the validated Helsinki chronic pain index (HCPI), was e-mailed to all owners whose dog was known to not have had surgical repair following treatment. The questionnaire inquired about duration of lameness following treatment, if their performance/sporting dog returned to sport, and if so at what level compared to prior to injury. To assess the dog’s current well-being, owners were asked to rate their dog’s quality of life in the last 7 days (excellent, very good, good, fair, poor) and were asked their opinion of their dog’s procedural outcome (excellent, good, fair, poor). Possible chronic pain was assessed with the validated HCPI that contained questions on the dog’s mood, lameness, and willingness to move, play, and jump. The HCPI contained 11 questions whose answers were later given a value (0–4), giving a total index score range of 0–44. Individual question scores of 0 and 1 indicate normal behavior and movement, where scores of 2, 3, and 4 indicate pain with increasing severity. Therefore, healthy dogs usually have an HCPI between 0 and 11, and dogs with chronic pain will have a score of 12–44. The HCPI has been validated, reliability tested, and responsiveness tested (42, 43) (Table 1).

**Table 1:** **Helsinki chronic pain index for veterinary use, total points: 36**.

Question asked	0 Points	1 Point	2 Points	3 Points	4 Points
Rate your dog’s mood	Very alert	Alert	Neither alert nor indifferent	Indifferent	Very indifferent
Rate your dog’s willingness to participate in play	Very willing	Willing	Reluctantly	Very reluctantly	Does not play at all
Rate your dog’s vocalization (audible complaining)	Never	Hardly ever	Sometimes	Often	Very often
Rate your dog’s willingness to walk	Very willing	Willingly	Reluctantly	Very reluctantly	Does not walk at all
Rate your dog’s willingness to trot	Very willing	Willingly	Reluctantly	Very reluctantly	Does not trot at all
Rate your dog’s willingness to gallop	Very willing	Willingly	Reluctantly	Very reluctantly	Does not gallop at all
Rate your dog’s willingness to jump (e.g., into car, onto sofa)	Very willing	Willingly	Reluctantly	Very reluctantly	Does not jump at all
Rate your dog’s ease in lying down	With great ease	Easily	Neither easily nor difficultly	With difficulty	With great difficulty
Rate your dog’s ease in rising from a lying position	With great ease	Easily	Neither easily nor difficultly	With difficulty	With great difficulty
Rate your dog’s ease of movement after long rest	With great ease	Easily	Neither easily nor difficultly	With difficulty	Very often/always difficulty
Rate your dog’s ease of movement after major activity or heavy exercise	With great ease	Easily	Neither easily nor difficultly	With difficulty	Very often/always difficulty

### Statistical Analysis

Data analyses were performed using a statistical software program (GraphPad Prism version 6.00, GraphPad Software, La Jolla, CA, USA). Gait analysis data were analyzed using a paired *t*-test that accounts for correlations between paired observations. A paired *t*-test will result in increased statistical power to detect a treatment effect when compared to an independent *t*-test. Outcome versus percent tear contingency data were analyzed using the Chi-square test for trend. Outcome versus use of a custom stifle orthotic contingency data were analyzed using the Fisher’s exact test. Outcome versus type of treatment (BMAC–PRP or ADPC–PRP) received contingency data were analyzed using a Fisher’s exact test. All the results were considered significant at *p* < 0.05.

## Results

### Signalment

In accordance with AAALAC International Rules of Accreditation, this study was performed with the approval of the VOSM Research Committee and with owner’s consent. All clients volunteered their dog for the study and provided written consent as required by Veterinary Orthopedic and Sports Medicine Group for every study participant. Medical records of 36 dogs diagnosed with an early partial (≤50%) CCL tear that were treated with BMSC–PRP or ADPC–PRP were reviewed. Ages ranged from 1 year to 9.5 years (mean 4.8 years, median 4.7 years), with 18 dogs being female (6 intact) and 18 dogs being male (12 intact). Body weights ranged from 4.8 to 60.5 kg (mean 25.9 kg, median 22.6 kg). Breeds included 10 (27.8%) Border Collies, 6 (16.7%) Labrador Retrievers, 3 (8.3%) Australian Shepherds, 2 (5.6%) Golden Retrievers, 2 (5.6%) Pembroke Welsh Corgis, 2 (5.6%) Shelties, and 1 (2.8%) of each of the following: Akbash, Basset Hound, Bull Terrier, Dutch Shepherd, German Shorthaired Pointer, Giant Schnauzer, Gordon Setter, Irish Setter, Keeshond, Mixed Breed, and Portuguese Water Dog. The majority of dogs were performance/sporting dogs [26 (72%) versus 10 (28%)] that were a companion dog only (Table 2). Duration of lameness ranged from 3 days to 4 years. Nineteen cases received BMAC–PRP for treatment, while 17 cases received ADPC–PRP. A custom stifle orthotic was used posttreatment in 14 dogs to prevent damage to the CCL during the recovery period.

**Table 2:** **Patient occupation breakdown**.

Occupation	Number of dogs
Companion	10
Performance/sport	26
Agility	15
Conformation	3
Flyball	3
Hunting	3
Herding	2
Obedience	2
Field trial	1
Search and rescue	1
Service dog	1

### Orthopedic Examination

Baseline orthopedic examination findings were available in 30 of the 36 dogs. A weight-bearing lameness and shortened stride was noted in 29 dogs, while 1 dog had a shortened stride only. On palpation, very mild effusion was present in the affected limb of 1 dog, mild effusion was present in 11 dogs, moderate effusion in 17 dogs, and 1 dog had no effusion. McMurray test for meniscal evaluation was negative in 29 dogs, while 1 dog was positive. Mild cranial drawer and tibial thrust were noted in flexion only in the affected limb in 23 dogs. Palpable evidence of stifle instability was absent in seven dogs.

### Radiographs

Baseline radiograph findings were available in 30 of the 36 dogs. Very mild joint effusion was noted in the affected limb of 3 dogs, mild effusion in 12 dogs, and moderate effusion in 14 dogs. One dog had no effusion present on radiographs. Osteoarthritis was also present on radiographs in the affected limb of five dogs. Joint effusion was noted in the contralateral limb in 15 dogs, with osteoarthritis being present in 1 (39).

### Objective Gait Analysis

**Figure 1:**
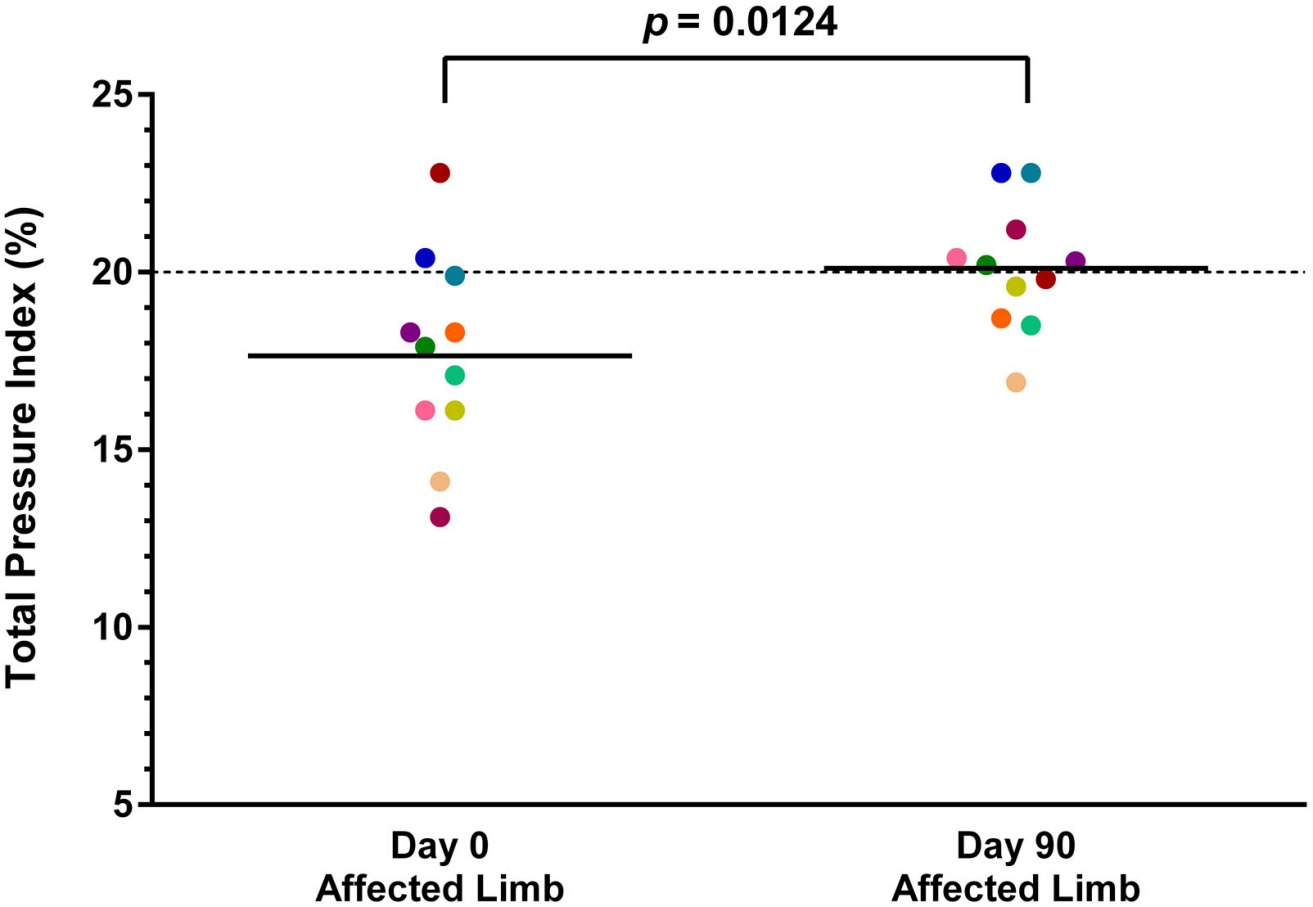
**A statistically significant difference was found between the treated limb total pressure index percent (TPI%) at day 0 and day 90 (*p* = 0.0124) (solid line, mean; dotted line, normal TPI%)**.

**Figure 2:**
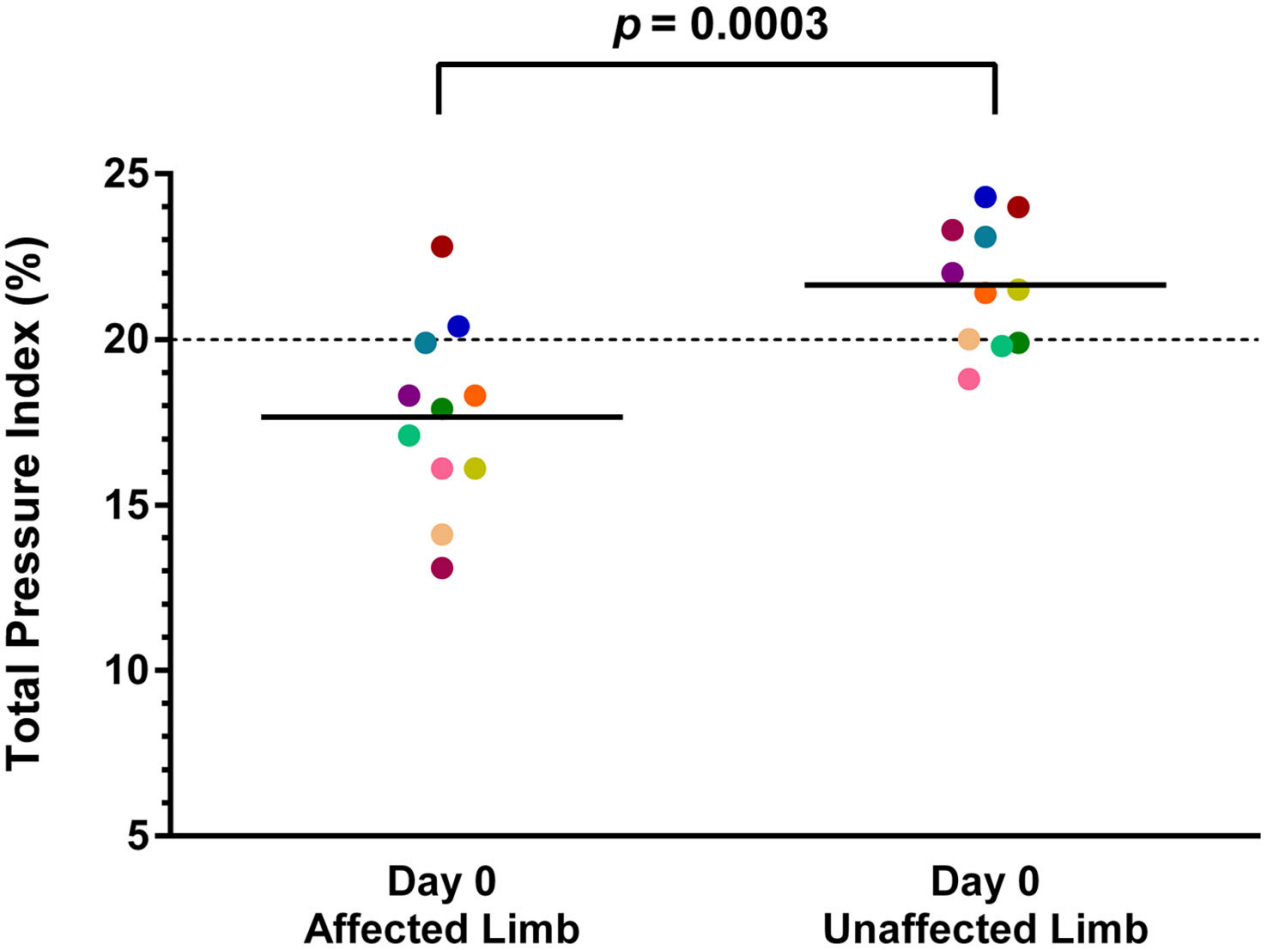
**A statistically significant difference was found between the treated limb and contralateral limb total pressure index percent (TPI%) at day 0 (*p* = 0.0003) (solid line, mean; dotted line, normal TPI%)**.

**Figure 3:**
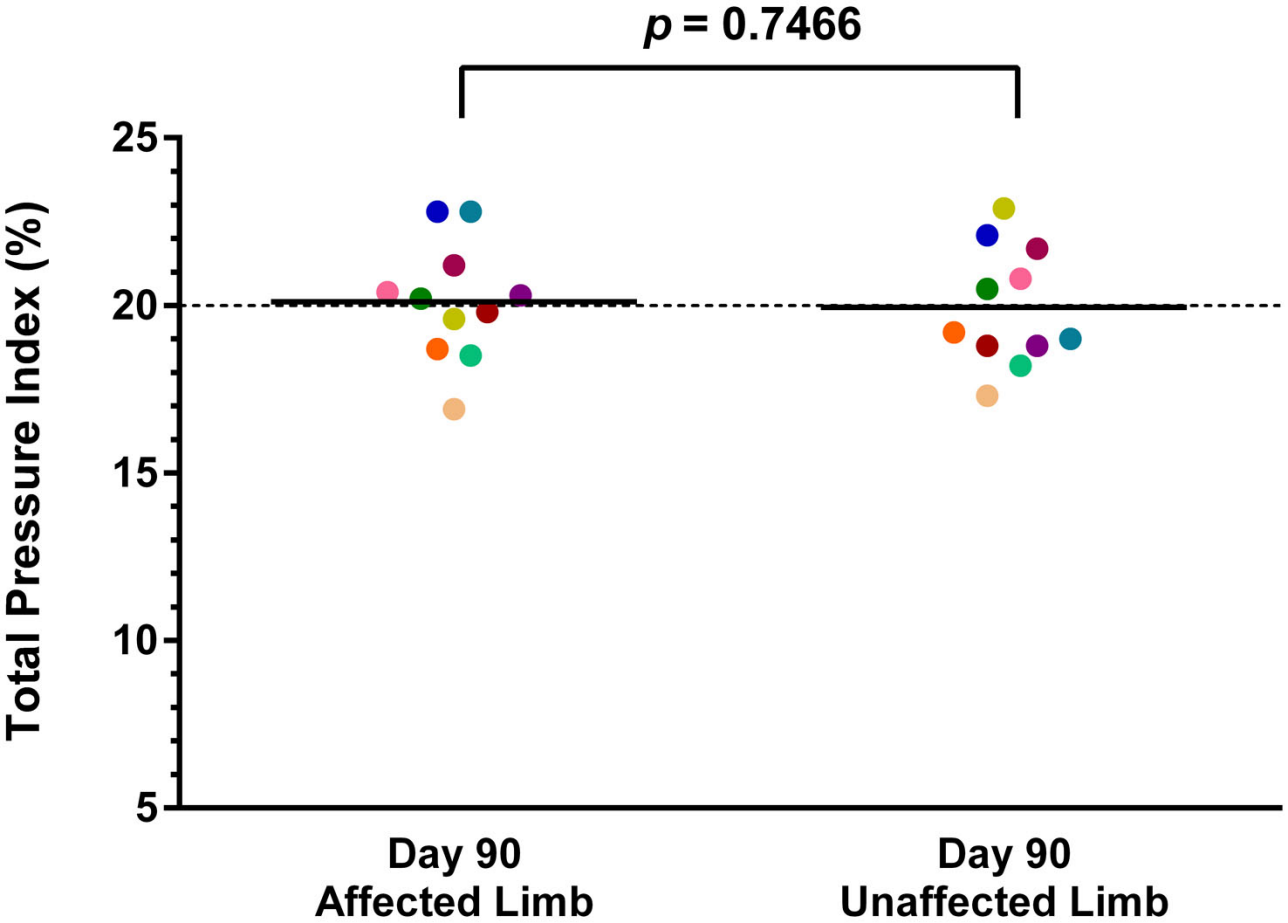
**No significant difference was found between the treated limb and contralateral limb total pressure index percent (TPI%) at day 90 (*p* = 0.7466) (solid line, mean; dotted line, normal TPI%)**.

Both baseline and day 90 posttreatment objective gait analyses were available on 11 of the 36 dogs. Mean baseline TPI% was 17.7% (median 17.9%, range 13.1–22.8%) in the affected limb and 21.7% (median 21.5%, range 18.8–24.3%) in the unaffected limb. At 90 days posttreatment, mean TPI% was 20.1% (median 20.2%, range 16.9–22.8%) in the treated limb and 19.9% (median 19.2%, range 17.3–22.9%) in the contralateral limb. The TPI% of the treated limb was compared to the TPI% of the contralateral limb at baseline and at 90 days posttreatment. The TPI% of the treated limb at day 0 was also compared to the TPI% of the treated limb at 90 days posttreatment. A statistically significant difference was found between the treated limb TPI% at day 0 and day 90 (Figure 1; *p* = 0.0124), and between the treated limb and contralateral limb TPI% at day 0 (Figure 2; *p* = 0.0003). No significant difference was found between the treated limb and contralateral limb TPI% at day 90 (Figure 3; *p* = 0.7466).

### Diagnostic Stifle Arthroscopy

**Figure 4:**
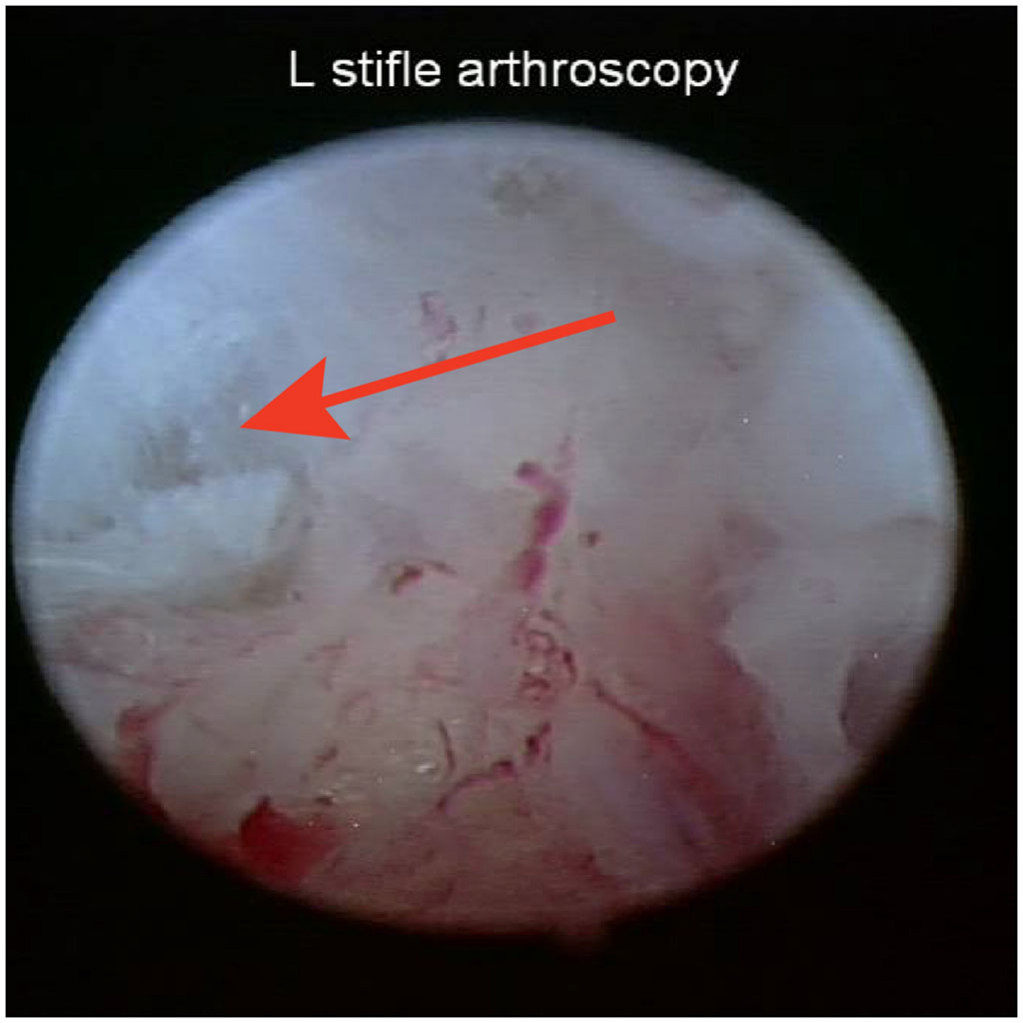
**Representative image of an early partial tear of the craniomedial band of the cranial cruciate ligament at the baseline stifle arthroscopy**.

Baseline diagnostic stifle arthroscopy findings were available on all 36 dogs. Diagnosis was confirmed and the CCL was found to be partially torn unilaterally in 35 dogs (13 left, 22 right), and bilaterally in 1 dog. The dog with bilateral partial CCL tears was omitted from the study, and the other 35 dogs were included in this study. A percent tear of the craniomedial band of the CCL was recorded for each dog. The high end was used to calculate descriptive statistics where a range was reported. Mild fraying of the craniomedial band of the CCL was reported in 4 of the 35 dogs. The percent tear of the craniomedial band of the CCL ranged from 5 to 50% (mean 20.8%, median 20%) in the remaining 31 dogs (Figure 4). The meniscus was found to be intact in 34 dogs, whereas 1 dog was found to have a bucket handle tear of the caudal pole of the medial meniscus, and a partial meniscectomy of the medial meniscus was performed. Degenerative joint disease (DJD) findings were recorded in 31 of the 35 dogs. Very mild DJD was reported in 2 dogs, mild DJD was reported in 4 dogs, moderate DJD in 2 dogs, and no DJD was found in the remaining 23 dogs.

**Figure 5:**
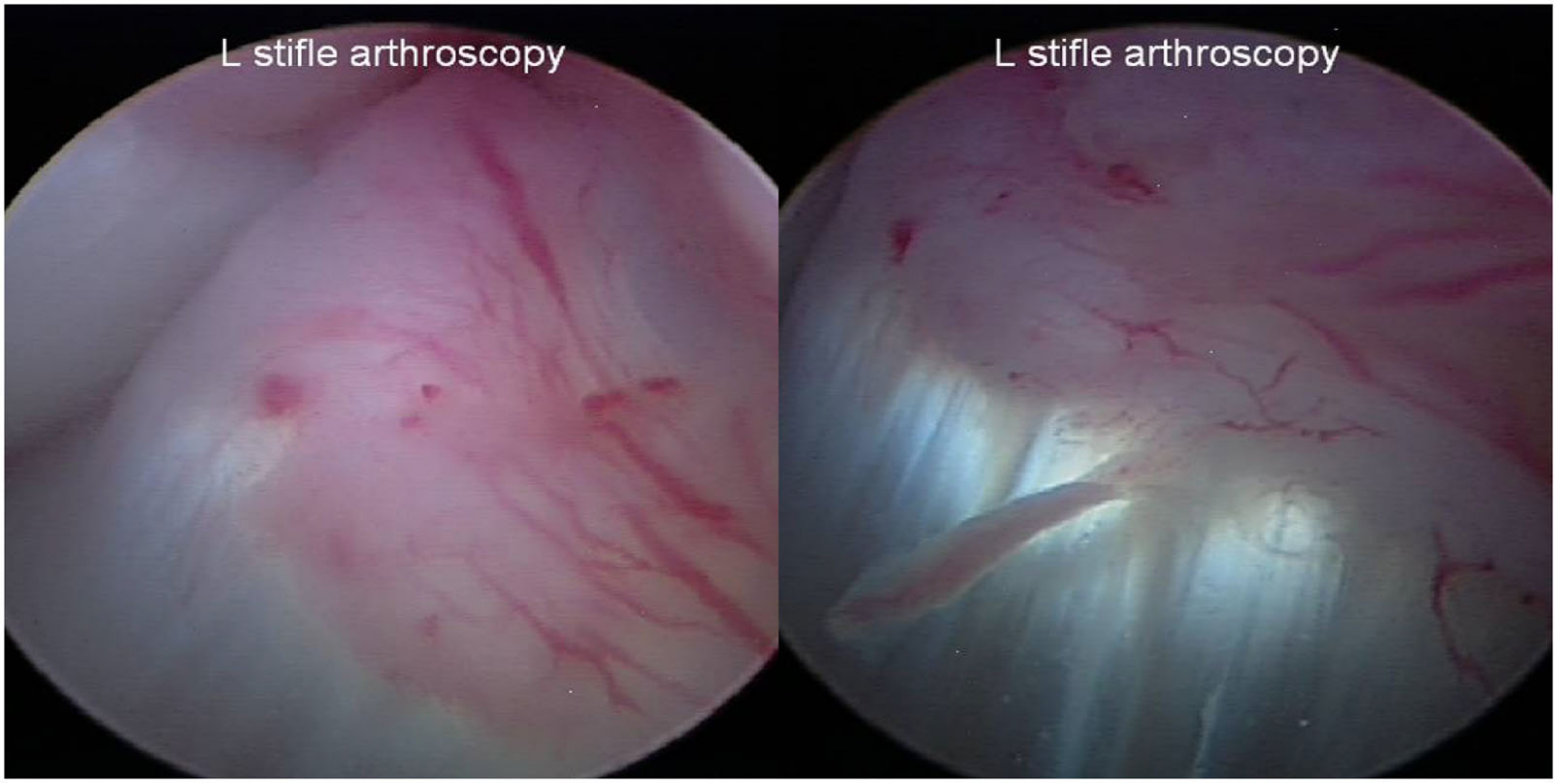
**Representative images of a fully intact cranial cruciate ligament with marked neovascularization and a normal fiber pattern with all previous regions of disruption healed at the day 90 posttreatment stifle arthroscopy**.

Stifle arthroscopy findings at 90 days posttreatment were available on 13 of the 35 dogs. In 9 of the 13 dogs, a fully intact CCL with marked neovascularization and a normal fiber pattern was found with all previous regions of disruption healed. It appeared that the blood supply to the region of CCL injury was coming from the genicular artery (Figure 5). One dog was found to have significantly improved by way of an intact CCL with minor fraying, compared to a 25% tear of the craniomedial band of the CCL at baseline, and received an additional injection. The dog was found to have normal range of motion and no discomfort, tibial thrust, or lameness 90 days after the second injection. The remaining three dogs were found to have a >50% tear (either progressive since baseline or completely ruptured), and a TPLO was performed at VOSM. Four additional dogs were reported by the owner to have had a TPLO performed outside of VOSM.

### Validated Functional Questionnaire

Questionnaires were sent to all owners whose dog was known to not have had surgical repair following treatment (29 dogs). Questionnaires were returned for 12 dogs (41%), 6 of which had received ADPC–PRP and 6 of which had received BMAC–PRP. Follow-up time between surgery and the questionnaire ranged from 0.5 to 2.9 years (mean 1.8 years, median 1.9 years). HCPI ranged from 0 to 17 (mean 4.4, median 1, standard deviation 6.3). Owners were asked how long their dog experienced lameness following treatment; the answers ranged from 0 to 12 months (mean 2.4 months, median 1.3 months). All 12 respondents believed their dog had an excellent (10) or very good (2) quality of life in the last 7 days and rated their dog’s procedural outcome as excellent (11) or good (1). Of the 12 questionnaires returned, 8 were reported to be performance/sporting dogs. According to the owner, seven of the eight dogs had returned to sport; the remaining dog had just begun a return to sport conditioning program 6 months posttreatment. Of the seven dogs that returned to sport, five dogs were reported to be competing at the same level compared to prior to injury, and two dogs were competing at a greater level compared to prior to injury (including faster times, higher jump height, and dropping bars less). According to the previously established VOSM Return to Agility Grading Scale (Table 3), of the four dogs that competed in agility, two dogs (50%) were grade 4 and two dogs (50%) were grade 5. Of the seven dogs that returned to sport, it was found that the greatest amount of time a dog had been competing since their procedure was 2.5 years.

**Table 3:** **Veterinary Orthopedic & Sports Medicine Group return to Agility Grading Scale**.

Description	Grade
Dog did not return to agility for reason unrelated to surgery	1
Dog was physically unable to return to agility	2
Dog returned to agility and performed worse than preoperative level	3
Dog returned to agility and performed at the preoperative level	4
Dog returned to agility and performed better than preoperative level	5

### Comparisons

The seven reported failures were found to have had a 50% (1), 25% (1), 20% (1), and 10% (4) tear at baseline. There was no association found between outcome and the percent tear reported at baseline (*p* = 0.5545). A custom stifle orthotic was used posttreatment in 14 dogs, with 4 of the reported failures having used an orthotic, and 3 failures not having used an orthotic. There was no association found between outcome and the use of an orthotic posttreatment (*p* = 0.5545). There were four reported failures that received ADPC–PRP treatment and three failures that received BMAC–PRP treatment. There was no association found between outcome and the treatment type received (*p* = 0.6843). All seven dogs that failed went on to have a TPLO procedure.

## Discussion

In this study, we investigated the use of autologous BMAC–PRP or ADPC–PRP combination for the treatment of early partial CCL tears in dogs and found that both provide promising potential for clinical use, which was supported by objective gait analysis, diagnostic arthroscopy, and validated functional questionnaire. Our results support the findings of previous studies in which MSCs engrafted to the CCL (27, 28) and when intra-articularly injected have been found to accelerate the healing of partial ACL tears in rats (30, 31). It is unknown if the MSCs used in our study engrafted in order to regenerate the CCL. Furthermore, our results demonstrate the clinical relevancy of these previous findings in dogs and provide further evidence to support clinical findings found in a previously reported case series in humans (32). Additionally, the questionnaire revealed that the greatest amount of time a dog had been competing since their procedure was 2.5 years, demonstrating that the CCL is able to withstand the continued rigor of sport.

The main limitations of this study are related to the nature of a retrospective study, including variation in treatments and rehabilitation therapy, loss to follow-up, and incomplete documentation. Most owners and patients whose data were collected for this study traveled from out of state for diagnosis and treatment, several being from across the country, thereby potentially contributing to the loss to follow-up and lack of follow-up findings. This element may also have played a role in the treatment type chosen by the owner. A greater return in questionnaires would be beneficial, thus positively or negatively affecting the outcomes associated with the survey, including the HCPI, the owner’s opinions on outcome, and the rate of dogs that returned to sport. Additionally, owner compliance to rehabilitation therapy recommendations was unknown. No associations that may provide a possible cause or correlation to treatment outcome could be deduced. Owner compliance to the strict at home confinement restrictions and guidelines given posttreatment are unknown and therefore may play a factor. Unfortunately, the exact stem cell numbers injected are unknown, which is a limitation of this study. However, previous validation work shows that there are approximately 5 million ADPC versus 30,000 BMAC (44). In spite of the large discrepancy in cell numbers between the ADPC and BMAC, it was determined that neither treatment was superior to the other in terms of outcome. Therefore, it appears that both treatment types possess the ability to regenerate the CCL; however, the cell number required still remains unknown. Additionally, difference in platelet concentration between the two systems used could affect outcome, and it is unclear what effect this had on outcomes for this study. Finally, because our standard of care is to utilize a stem cell and PRP combination, it is still unknown if stem cells or PRP alone could produce the same results.

In conclusion, our study provides promise to the use of autologous BMAC–PRP or ADPC–PRP combination as an alternative treatment option. However, further studies are needed to determine if they are reproducible in a well-populated randomized, blinded, and controlled trial, reporting data from BMAC and ADPC separately. Cell counts and biomarker assays should be included in future studies. Clinical findings and a validated functional questionnaire should be documented before and after treatment.

## Ethics Statement

This was a retrospective study and only gathered data from previously performed treatments on client-owned dogs. In accordance with AAALAC International Rules of Accreditation, this study was performed with the approval of the VOSM Research Committee and with owner consent. All clients volunteered their dog for the study and provided written consent as required by Veterinary Orthopedic and Sports Medicine Group for every study participant. All clients volunteered their dog for the study and provided written consent as required by Veterinary Orthopedic and Sports Medicine Group for every study participant.

## Author Contributions

All the authors contributed to the conception or design of the work, drafting and revising, and final approval of the version to be published.

## Conflict of Interest Statement

Dr. SC is a consultant for Companion Regenerative Therapies. The authors report no other conflicts of interest.

## Abbreviations

CCL, cranial cruciate ligament; TPLO, tibial plateau leveling osteotomy; TPI%, total pressure index percent; ACL, anterior cruciate ligament; MSC, mesenchymal stem cell; BMAC, bone marrow aspirate concentrate; ADPC, adipose-derived progenitor cells; PRP, platelet-rich plasma; BM-MSC, bone marrow-derived mesenchymal stem cells.
